# Machine Learning of Infant Spontaneous Movements for the Early Prediction of Cerebral Palsy: A Multi-Site Cohort Study

**DOI:** 10.3390/jcm9010005

**Published:** 2019-12-18

**Authors:** Espen A. F. Ihlen, Ragnhild Støen, Lynn Boswell, Raye-Ann de Regnier, Toril Fjørtoft, Deborah Gaebler-Spira, Cathrine Labori, Marianne C. Loennecken, Michael E. Msall, Unn I. Möinichen, Colleen Peyton, Michael D. Schreiber, Inger E. Silberg, Nils T. Songstad, Randi T. Vågen, Gunn K. Øberg, Lars Adde

**Affiliations:** 1 Department of Neuromedicine and Movement Science, Norwegian University of Science and Technology, 7491 Trondheim, Norway; espen.ihlen@ntnu.no; 2 Department of Neonatology, St. Olavs Hospital, Trondheim University Hospital, 7006 Trondheim, Norway; ragnhild.stoen@ntnu.no; 3 Department of Clinical and Molecular Medicine, Norwegian University of Science and Technology, 7491 Trondheim, Norway; Toril.Fjortoft@stolav.no; 4 Ann and Robert H Lurie Children’s Hospital of Chicago, Chicago, IL 60611, USA; LBoswell@luriechildrens.org (L.B.); r-deregnier@northwestern.edu (R.-A.d.R.); 5 Feinberg School of Medicine, Northwestern University, Chicago, IL 60611, USA; dgaebler@sralab.org (D.G.-S.); colleen.peyton1@northwestern.edu (C.P.); 6 Clinic of Clinical Services, St. Olavs Hospital, Trondheim University Hospital, 7006 Trondheim, Norway; randi.tynes.vagen@stolav.no; 7 Shirley Ryan AbilityLab, Chicago, IL 60611, USA; 8 Department of Clinical Therapeutic Services, University Hospital of North Norway, 9038 Tromsø, Norway; Cathrine.Labori@unn.no (C.L.); gunn.kristin.oeberg@uit.no (G.K.Ø.); 9 Department of Pediatrics, Division of Paediatric and Adolescent Medicine, Oslo University Hospital, 0372 Oslo, Norway; Marianne.loennecken@gmail.com (M.C.L.); umoinich@ous-hf.no (U.I.M.); isilberg@ous-hf.no (I.E.S.); 10 University of Chicago Medicine, Comer Children’s Hospital, Section of Developmental and Behavioral Pediatrics, Chicago, IL 60637, USA; mmsall@peds.bsd.uchicago.edu (M.E.M.); mschreiber@peds.bsd.uchicago.edu (M.D.S.); 11 University of Chicago, Kennedy Research Center on Intellectual and Neurodevelopmental Disabilities, Chicago, IL 60637, USA; 12 Department of Pediatrics, Comer Children’s Hospital, Department of Physical Therapy and Human Movement Science, Chicago, IL 60637, USA; 13 Department of Pediatrics and Adolescent Medicine, University Hospital of North Norway, 9038 Tromsø, Norway; Nils.Thomas.Songstad@unn.no; 14 Department of Health and Care Sciences, Faculty of Health Sciences, UiT- The Arctic University of Norway, 9019 Tromsø, Norway

**Keywords:** cerebral palsy, premature infants, general movement assessment, machine learning

## Abstract

Background: Early identification of cerebral palsy (CP) during infancy will provide opportunities for early therapies and treatments. The aim of the present study was to present a novel machine-learning model, the Computer-based Infant Movement Assessment (CIMA) model, for clinically feasible early CP prediction based on infant video recordings. Methods: The CIMA model was designed to assess the proportion (%) of CP risk-related movements using a time–frequency decomposition of the movement trajectories of the infant’s body parts. The CIMA model was developed and tested on video recordings from a cohort of 377 high-risk infants at 9–15 weeks corrected age to predict CP status and motor function (ambulatory vs. non-ambulatory) at mean 3.7 years age. The performance of the model was compared with results of the general movement assessment (GMA) and neonatal imaging. Results: The CIMA model had sensitivity (92.7%) and specificity (81.6%), which was comparable to observational GMA or neonatal cerebral imaging for the prediction of CP. Infants later found to have non-ambulatory CP had significantly more CP risk-related movements (median: 92.8%, *p* = 0.02) compared with those with ambulatory CP (median: 72.7%). Conclusion: The CIMA model may be a clinically feasible alternative to observational GMA.

## 1. Introduction

Cerebral palsy (CP) encompasses a heterogeneous group of motor impairments in childhood that affect the development of movement and posture, causing activity limitation [1]. The prevalence of CP is 2.1 cases per 1000 in high-income countries and occurs in up to 10% of infants at highest risk [2]. CP is a diagnosis based on clinical and neurological signs and is typically determined between age 12 and 24 months [3]. Earlier identification of infants with CP would improve access to community services [4], improve well-being for parents [5] and provide social and economic support for those infants and families in need of care [6]. Early identification would also facilitate earlier onset of therapies and treatments in the period when plasticity of the infant brain is at its highest [7]. Today, the most accurate risk assessments of CP in infants before 5 months of age are the observational general movement assessment (GMA) and cerebral imaging [3]. However, these risk assessments are either based on qualitative perception, requiring considerable training and clinical expertise (GMA), or demand highly expensive equipment (cerebral imaging) [8]. Thus, research on low-cost alternatives for early risk assessment of CP based on automatic and objective detection of infant spontaneous movements has rapidly increased the last two decades [9,10]. 

Automatic detection of infant spontaneous movements is based on several types of technology including 3D motion capture, inertial sensors, and video recordings [9]. The most clinically feasible technology is a video recording, which is non-intrusive, not dependent on body worn reflective markers or inertial sensors, and available in most clinical and home-based settings using commercially available video and smartphone cameras [10]. Because of the clinical use of observational GMA, large databases of video recordings and CP outcomes are becoming available. These serve as rich sources of data that are important for the generation of robust prediction models based on machine learning. Furthermore, novel methods within machine learning and computer vision have improved possibilities for automated infant motion tracking and facilitated the further development of a computer-based assessment of infant movement kinematics [11]. Several studies have predicted CP based on an automatic movement assessment from infant video recordings with performance comparable to observational GMA [12,13,14,15,16]. A summary of the results of these studies, the methods used, and sample sizes are shown in Table 1. Kanemura et al. [17] also found that infants developing CP had higher average velocity and jerky movements of the legs. However, this study did not report the sensitivity and specificity of the method predicting CP.

Despite promising results, previous studies using automatic assessment of spontaneous infant movements have several fundamental shortcomings: First, all studies, except the study of Orlandi et al. [16], are based on convenience samples that do not reflect typical clinical cohorts. Second, study samples are small (*N* = 13–16) in terms of number of children with CP, and it is uncertain whether the prediction models in these studies have external validity for application in a representative population of high-risk infants. Third, the construct validity of the movement features included in previous prediction models is questionable. Observational GMA defines that infant spontaneous movements have complexity denoted by a flow of changes in the movement direction of the participating body parts and variation across time where the infant explores the movement possibilities that the body offers. These spatial and temporal changes in movements are tightly intertwined [18]. The spatial and temporal changes in these movement features are difficult to represent as a single feature across the entire video recording, as was carried out in a previous study using the standard deviation of the center of motion [12]. Our hypothesis is that complex and variable spontaneous movements could be characterized by multiple features of temporal modulation in movement frequencies and covariation that will outperform single features, and that spatial and temporal changes in infant movement can be assessed by dividing the video recording into smaller movement periods to obtain a percentage (i.e., proportion) of periods with CP risk-related movements. Furthermore, previous studies have not investigated the relationship between CP prediction models and gross motor function in children with CP. These are important elements to ensure the construct validity and, consequently, the feasibility of the CP prediction model for clinical decision support. 

The aim of the present study was to present a novel machine-learning model, the Computer-based Infant Movement Assessment (CIMA) model, for clinically feasible early CP prediction and for the prediction of ambulatory (gross motor function classification scale (GMFCS I–III) versus non-ambulatory function (GMFCS IV–V) in children with CP. 

## 2. Experimental Section

### 2.1. Study Participants

This study is part of a multi-center, observational study on early CP prediction in high-risk infants. Four hundred and fifty infants admitted to one of five participating level III–IV Neonatal Infant Care Units (NICU) in Norway or the United States were enrolled at discharge from the NICU based on extreme prematurity, neonatal neurologic abnormalities, cardiac surgery or medical complexity. Video recordings during the fidgety movements period were taken according to Prechtl’s methodology for observation of general movements, and the GMA results for early CP prediction are presented in a different paper [19]. In the study arm presented here, the aim was to develop a novel machine-learning algorithm for early CP prediction based on the same videos.

**Figure 1:**
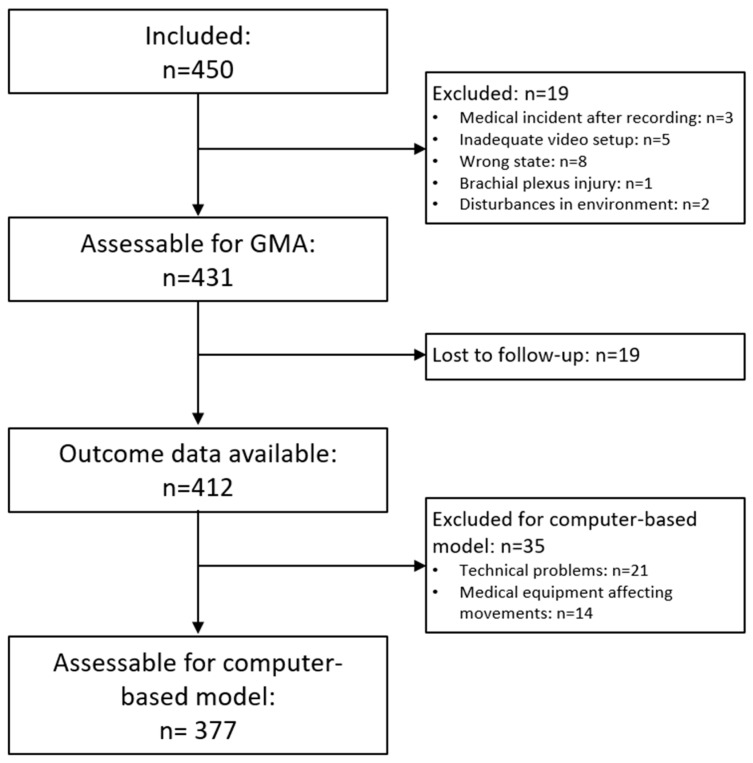
Flow-chart of exclusion of video recordings for the development and testing of the Computer-based Infant Movement Assessment (CIMA) model.

In total, 377 infants constituted the study sample after exclusion of video recordings (see Figure 1). The median length of the included video recordings was 5 min (range: 1–5 min) and the mean age of the infants at recording was 12 (9–15) weeks corrected age (CA). For details on the clinical characteristics and neonatal risk factors of the 377 included infants, see Appendix A.

### 2.2. The Computer-Based Infant Movement Assessment (CIMA) Model

**Figure 2:**
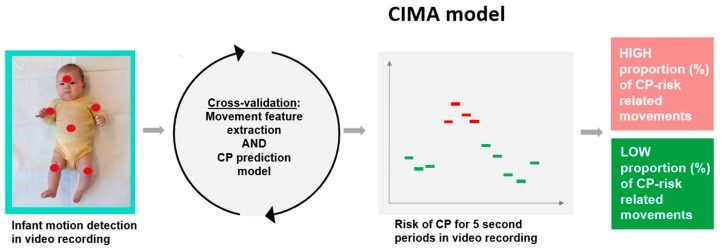
Steps of the CIMA model. First, infant movements are detected by motion tracking of six body parts (head, trunk, arms, and legs) in the video. Second, features for the movement frequencies, amplitude, and covariation of the different body parts are extracted from the body part movement trajectories and used in the CP prediction model. The CP prediction model identifies 5 second periods with CP risk-related movements. Finally, the proportion (%) of periods with CP risk-related movements typically found in infants with CP is summarized and communicated as a CP risk indicator.

The goal for the development of the CIMA model was to improve the early risk assessment of CP in high-risk infants before 5 months post-term age. Figure 2 summarizes the steps of the CIMA model. 

#### 2.2.1. Infant Motion Detection in Video Recording

Infants were video recorded during active wakefulness when in a comfortable state at 9 to 15 weeks CA using a standardized set-up, and in cases of more than one available video, the one closest to 12 weeks CA was selected. A commercially available digital video camera (Sanyo VPC-HD2000, SANYO Electric Co, Ltd., Osaka, Japan) was used. The processing of the video recording contained five steps: video screening, preprocessing, pixel tracking using large displacement optical flow, segmentation of six body parts, and extraction of vertical and horizontal coordinates of body part’s movements. All videos were cropped so that only the infant was visible in the video. Large displacement optical flow was used to track pixel movements and a manual annotation was performed on each 500 frames to identify the pixel center of six parts of the infant’s body—arms, legs, head, and torso. Two research assistants without any expertise in infant spontaneous movements performed the manual annotation. Technical details of the infant motion tracker method are described elsewhere [20]. 

#### 2.2.2. Movement Feature Extraction 

To quantify the temporal variation in body part movement frequencies, amplitude, and covariations, the horizontal (x) and vertical (y) coordinates of the pixel center of the six body parts was decomposed into the time–frequency domain by multivariate empirical mode decomposition (MEMD) and Hilbert–Huang transformation [21,22]. In contrast to the fast Fourier transformation and wavelet methods chosen in previous studies [13,14,15], the movement components defined by MEMD have the potential to reflect intrinsic properties of the infant movement dynamics. Technical details of the procedure are described in Appendix B. The body parts’ mean movement frequency, amplitude, and covariation was computed for 5 second non-overlapping time periods and finally resulted in a set of 990 features describing all the infant’s movement repertoire in each 5 second period. 

#### 2.2.3. CP Prediction Model and Validity of the Model

Each 5 second period in the video was labeled as CP or non-CP according to the child’s CP status diagnosed according to the decision tree published by the Surveillance of cerebral palsy in Europe (SCPE, [23]). In total, 1898 periods were available from videos of children with CP and 18,321 periods from videos of children without CP. A partial least square (PLS) regression with a backward feature selection was performed to select features that predicted CP from the large set of 990 movement features without overfitting the model [24]. The selected movement features in each 5 second period were clustered into 5 composite scores which were used in a linear discriminative analysis (LDA) to classify movements typically found in children with or without CP (Appendix C) [25]. To avoid overfitting of the CIMA model, the dataset was divided into training, validation, and test sets in a double cross-validation procedure (Appendix D). The final CIMA model classified each 5 second period with either 0 or 1 according to the absence or presence of CP risk-related movements. The final risk classification was averaged across each video recording defining the proportion (%) of periods with CP risk-related movements. A decision threshold of 50% was set to decide whether the video represented an infant with overall absence or presence of CP risk-related movements.

### 2.3. Observational GMA, Cerebral Imaging, Cerebral Palsy and Gross Motor Function

*Observational GMA* was carried out on the same video recordings as the CIMA model according to the Prechtl approach [26]. Two experienced and certified GMA observers (LA and TF) who were blinded to the clinical history of the infants performed all assessments. In case of disagreement, the observers re-assessed the video together and reached consensus. Fidgety movements (FM) were classified as absent (FM−; *n* = 57/15%), sporadic (FM−/+; *n* = 29/8%), intermittent (FM+; *n* = 235/62%), continual (FM++; *n* = 49/13%) according to their presence and length of interspersed pauses [27], or as exaggerated (FMa, *n* = 7/1.9%) if excessive in amplitude and speed.

*Cerebral imaging* (cerebral ultrasound (cUS) and magnetic resonance imaging (MRI)) was carried out for clinical purposes following each hospital’s guidelines, and a central classification of the results into normal/mildly abnormal or moderately/severely abnormal was carried out based on the local written reports. Lesions known to be associated with later CP were classified as abnormal, and milder abnormalities not associated with later CP were classified as normal (for details about imaging results, see [19]). 

*Assessment of cerebral palsy and ambulatory motor function:* CP was diagnosed according to the decision tree published by the Surveillance of cerebral palsy in Europe (SCPE, [23]). The CP diagnosis was performed by pediatricians who were unaware of the outcome of GMA classification and CIMA model. Gross motor function was classified using the Gross Motor Function Classification System (GMFCS) [28,29]. Forty-one (11%) of 377 included infants had CP with corresponding GMFCS status at follow-up at mean age 3.7 years (SD 0.95; range 1.2–6 years). The prevalence of CP subtypes and GMFCS levels for the 41 infants developing CP are shown in Table 2 below.

### 2.4. Statistics of the Outcome of the CIMA Model

The ability of the CIMA model to predict CP was assessed by sensitivity, specificity, positive and negative predictive values (PPV and NPV) and area under receive operating characteristic curve (AUC). The performance of the CIMA model was compared with the performance of the observational GMA and cerebral imaging (cUS and MRI) [19] in addition to the automated method previously presented by our group using the variation of the spatial center of motion (C_SD_) [12,30]. Kruskal–Wallis’s test with the post hoc Wilcoxon rank-sum test including Bonferroni correction assessed the significance of the difference in the proportion of CP risk-related movements between the different FM categories assessed by the observational GMA. The Wilcoxon rank-sum test was also used to assess the significance of the difference in the proportion of CP risk-related movements between infants developing CP with GMFCS I, II, or III (i.e., ambulatory CP) and those developing CP with GMFCS IV or V (i.e., non-ambulatory CP). All analyses and statistics were performed in Matlab 2018a and *p*-values below 0.05 were considered statistically significant.

## 3. Results

### Proportion of Periods with CP Risk-Related Movements, CP Status and Gross Motor Function

**Figure 3:**
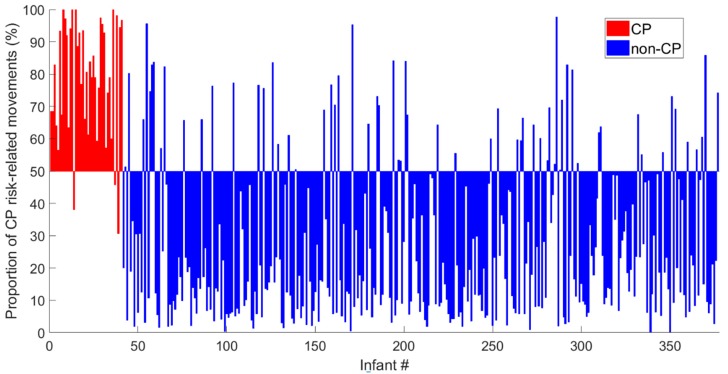
Each bar represents the proportion (%) of periods with CP risk-related movements represented in the video recordings of each of the 377 infants. The bars are centered around the decision threshold of 50% (horizontal line) for increased risk of CP. The red bars are from infants with confirmed CP diagnosis, whereas the blue bars represent the infants with a confirmed non-CP diagnosis.

Figure 3 shows the proportion of periods with CP risk-related movements identified in each of the video recordings of the 377 infants. Three (7.3%) of 41 children with a confirmed CP diagnosis had a proportion of periods with CP risk-related movements below 50% (false negative; red bars below the horizontal line in Figure 3). Sixty-two (18.5%) of 336 infants who did not develop CP had a proportion of CP risk-related movements above 50% (false positive; blue bars above the horizontal line in Figure 3). Table 3 shows the predictive values for the current CIMA model, observational GMA [19] and neuroimaging results [19] and the variation of the spatial center of motion (C_SD_). The CIMA model had the best sensitivity, NPV and AUC. However, the specificity and PPV were slightly lower than for the GMA and neuroimaging results. The statistics in Table 3 are dependent on a decision threshold of 50%. The ROC curve and alternative thresholds are provided in Appendix E and cross-tables and mean square contingency coefficients are provided in Appendix F.

**Figure 4:**
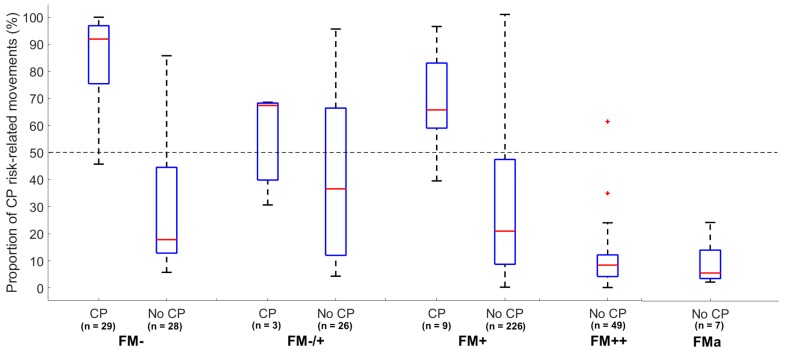
Boxplot of the proportion of periods with CP risk-related movements assessed by the CIMA model (y-axis) and temporal organization of FMs assessed by observational GMA (x-axis) according to CP outcome. The red line indicates the median and blue box the interquartile range. The whiskers in dashed lines are 1.5 times the interquartile range and cover 99.3% of the data if normally distributed. Outliers are marked as red crosses. The horizontal dashed line represents a decision threshold of 50% for the CIMA model. FM− = absent FM; FM−/+ = sporadic FM; FM+ = intermittent FM; FM++ = continual FM; FMa = FM with exaggerated speed and amplitude.

The proportion of periods with CP risk-related movements showed a significant relationship with the FM classification by the observational GMA (*p* < 0.01, Figure 4). In the group of infants with absent FMs, a significantly higher proportion of CP risk-related movements (median: 92%, *p* < 0.00001) were seen in the infants later diagnosed with CP when compared with those without CP (median: 18.6%) (see boxplots in Figure 4). Within the group of infants later diagnosed with CP, the infants with intermittent fidgety movements (FM+) had a significantly lower proportion of risk-related movements (median: 63.5%, *p* = 0.009) compared with the infants with absent fidgety movements (FM−). The infants with continual fidgety movements (FM++) had a low proportion of periods of risk-related movements (median: 8.5%), with little intra-group variation, compared with infants with absent, sporadic or intermittent FMs (Figure 4). 

Among the 41 children with CP, the proportion of periods with CP risk-related movements was significantly higher in those with GMFCS IV–V (non-ambulatory function) compared to those with GMFCS I–III (ambulatory function) (median: 92.8%, IQR: [75.0%, 97.2%] vs. median: 72.7%, IQR: [60.6%, 83.3%]; *p* = 0.02). 

## 4. Discussion

This study presents a novel machine-learning model, the CIMA model, for the early prediction of CP with an accuracy comparable to the General Movement Assessment (GMA) and neonatal cerebral imaging. Furthermore, the CIMA model differentiated children with ambulatory CP from those with non-ambulatory CP. These findings motivate the further development of a clinical decision support system based on video recordings and machine-learning assessment that can easily be applied for screening of high-risk infants. 

In the present study, the CIMA model was developed based on CP outcome. This is in contrast to others who have presented machine-learning models for automated CP prediction based on the identification of abnormal general movements and absence of FMs [9]. The video recordings in the present study were performed during the fidgety movements period, making it likely that the CIMA model captures some of the features which are typical for FM. Hence, the selected features in the CIMA model (i.e., movement covariation, frequencies and amplitudes) have the potential to reflect complexity and variability of the infant spontaneous movements which is typical for FM [27]. Both CIMA and GMA deliver a high number of false positives, but the assessments are weakly correlated with *r* = 0.24 to 0.30 (see Appendix F for details). The false positive cases of the CIMA method are mainly in the intermittent FM category (FM+), whereas the false positive of the GMA is, by definition, in the absent and the sporadic FM category (FM−/+ and FM+). The CIMA model identified children without CP who were classified with absence of FMs (i.e., FM−) with a low proportion of CP risk-related movements. These results suggest that the CIMA model and GMA identifies different false positive cases and may identify different features of infant spontaneous movements. Thus, machine-learning approaches, like the CIMA model, could be used to detect false positive cases within the group of infants with absence of FMs. Further research could relate the movements features used in the CIMA model to the different motor phenotypes recently suggested for infants developing CP in order to gain a deeper knowledge of the appearance of false positive cases in the CIMA model [31]. 

The ability of the CIMA model to predict ambulatory versus non-ambulatory function in children with CP suggests a continuum in the proportion (%) of periods with CP risk-related movements, which is related to later motor function. However, the present CIMA model cannot reveal how the chosen movement features change according to later motor function. For the time being, we can, therefore, only speculate that reduced covariation between body parts and reduced variation in movement frequencies and amplitudes are typical for infants who develop CP and that the same movement features are related to the severity of CP.

The proportion (%) of time periods with abnormal movements identified by the CIMA model was shown to outperform the CP prediction ability of the standard deviation of the center of motion (C_SD_) used in several previous computer-based studies by our group [12,30]. The previously developed C_SD_ was based on a frame differencing method which may be susceptible to differences in contrasts, light, and infant clothing, which may vary more in this larger multi-site cohort of infants. Furthermore, as the sample size and heterogeneity of children with CP increase, it becomes more challenging for a single predefined feature, such as C_SD_, to contain information of various characteristics of the infant movement repertoire relevant for a clinical outcome such as CP. Thus, we argue that it is likely that the predictive performance of other suggested single features such as relative movement frequency [15] and mean and minimum velocity [30] will potentially decay in larger multi-site populations of high-risk infants. The performance of the presented CIMA model suggests that overall variables, such as the proportion (%) of periods with CP risk-related movements, should be based on a cluster of movement features rather than single “key” features. 

The CIMA model has several clinical and methodological limitations. First, the large distance optical flow method is not fully automatic and requires manual annotation. Thus, even though the CIMA model could be a clinically feasible alternative to observational GMA, additional resources for manual annotation are necessary at this point. Furthermore, the horizontal and vertical coordinates of the pixel centers of the six labeled infant body parts are not directly related to biomechanical features such as the joint center position or the body part’s center of mass. Consequently, the present infant movement tracker based on large distance optical flow does not provide accurate biomechanical descriptors of the infant movements. Thus, the selected features and PLS regression components of the CIMA model will be dependent on the specific motion tracker system used. Further studies should emphasize on developing fully automated movement tracker technology able to identify joint center position and the body segment’s center of mass (i.e., full biomechanical 2D model), which will have the potential to identify specific and definable biomarkers of later motor impairments. Advancement in computer vision and the development of deep convolutional neural networks make it possible to identify joint centers and body segment position with high precision [11,32]. Such a movement tracker will generate universal pools of biomechanical descriptors for the CIMA model that are not dependent on manual annotations and the choice of movement assessment technology (e.g., 3D motion capture and inertial body worn sensors). 

Secondly, the CIMA model is trained on video recordings from a standardized camera setup with a static mounted camera [12]. To improve clinical feasibility, the CIMA model should be trained on video recordings from hand-held smartphone cameras. Thus, a fully automated movement tracker system suggested above could include filters and post-processing procedures to remove motion artifacts of hand-held smartphone recordings [11]. This will integrate the CIMA model into future app-based platforms for clinical decision support.

Thirdly, the present model was created from 5 second non-overlapping time periods. It is highly unlikely that all 5 second periods within a video recording contain infant movements related to risk of CP outcome. As an example, infants with or without CP may be quiet with little movements within a 5 second time period. These time periods containing only short movement sequences will get different CIMA model labels according to the CP outcome. Thus, the labeling of the short periods may contribute to noise and, consequently, this may affect the estimated percentage (%) of periods with CP risk-related movements. A remedy for these limitations is to use the classification score provided by linear discriminative analysis to weight the influence of each time interval. This solution was implemented in our study but did not change the reported overall performance of the CIMA model. 

Fourth, the present study did not provide test–retest reliability of the proportion of CP risk-related movements. The intra-session test-reliability of observational GMA is reported to be high [33]. Further test–retest reliability studies are necessary before concluding that the CIMA model is a clinically feasible alternative to observational GMA. 

Finally, the present study should be replicated on new samples of high-risk infants to assess the external validity of the CIMA model. The present multi-center study comprised a heterogenous selection of infants (shown in Appendix A, Table A1). The predictive values of a specific method will differ based on the prevalence of the outcome, and this should be taken into consideration in the interpretation of the results. The international community of infant movement assessment should collaborate on generating larger databases of infant video data working as a foundation for the development of more robust machine-learning algorithms for the classification of infant motor repertoire and the prediction of later motor impairments. The investigation of neurophysiological correlates (functional magnetic resonance imaging (fMRI), ultrasound, electroencephalography (EEG) and magnetic resonance imaging (MRI)) to the outcome of the CIMA model is also an important direction of future research to improve the model’s construct validity and establish new biomarkers of later motor impairments. 

## 5. Conclusions

This study presents a novel machine-learning model, called the CIMA model, which predicts CP in a large cohort of high-risk infants with an accuracy comparable to the observational General Movement Assessment (GMA) and neonatal cerebral imaging. The model also differentiated between ambulatory and non-ambulatory CP. Movement features assessing covariation between body parts and temporal modulation in movement frequencies and amplitudes were used in the CIMA model. The present adds to developing a clinical decision support system based on video recordings and machine-learning models that can easily be applied for screening of high-risk infants. 

## Figures and Tables

**Table 1:** Summary of results in previous studies for the prediction of cerebral palsy (CP) with video-based automated infant movement analysis.

Study	Sample Size ^1^	Sens. (%)	Spec. (%)	Acc (%)	Features
Adde [12]	30 (13)	85	88	88 *	C_SD_, QoM
Rahmati [13]	78 (14)	50	95	87	FFT features
Rahmati [14]	78 (14)	86	92	91	FFT features
Stahl [15]	82 (15)	85	95	94	Wavelet features
Orlandi [16]	127 (16)	44	99	92	FFT/time features

^1^ Sample size and number of infants with later CP diagnosis in parenthesis (..). * Value is area under receiver operating characteristic (ROC) curve. FFT = fast Fourier transformation (i.e., amplitude and frequency of infant movements); C_SD_ = standard deviation of the center of motion; QoM = quantity-of-motion.

**Table 2:** CP subtype and gross motor function in children with CP.

CP Status	*N* (%)
CP subtype *	
Unilateral spastic	8 (20)
Bilateral spastic	25 (61)
Dyskinetic	5 (12)
Ataxic	1 (2)
**Gross motor function (GMFCS)**	
GMFCS I	11 (27)
GMFCS II	3 (7)
GMFCS III	6 (15)
GMFCS IV	10 (24)
GMFCS V	11 (27)

* CP subtype was available in all but two of the 41 children with CP. GMFCS = Gross Motor Function Classification System.

**Table 3:** The sensitivity, specificity, positive and negative predictive values and area under the curve (AUC) with 95% confidence intervals in brackets for the prediction of CP.

Method	Sens. (%)	Spec. (%)	PPV (%)	NPV (%)	AUC *
CIMA	92.7 [80.1, 98.5]	81.6 [77.0, 85.5]	38.0 [32.5, 43.8]	98.9 [96.8, 99.6]	0.87 [0.81, 0.91]
GMA [19]	76.2 [60.6, 88.0]	82.4 [78.1, 86.2]	33.3 [27.4, 39.8]	96.8 [94.6, 98.1]	0.82 [0.78, 0.85]
Imaging [19]	81.0 [65.9, 91.4]	85.3 [81.2, 88.8]	39.1 [32.5, 46.1]	97.5 [95.4, 98.6]	0.85 [0.81, 0.88]
C_SD_	56.1 [39.8, 71.5]	58.6 [53.2, 64.0]	14.2 [10.9, 18.6]	91.6 [88.5, 94.0]	0.56 [0.48, 0.64]

* Values for GMA and Imaging is accuracy reported in Støen et al. [19]. PPV = positive predictive value; NPV = negative predictive value; GMA = General Movement Assessment.

**Figure A1:**
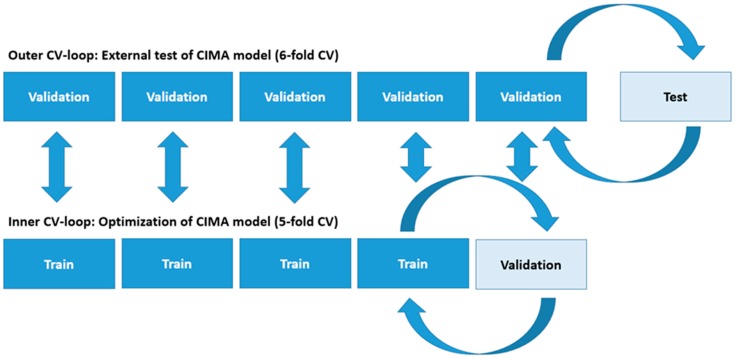
Schematic illustration of the cross-validation (CV) procedure used to validate the CIMA model.

**Figure A2:**
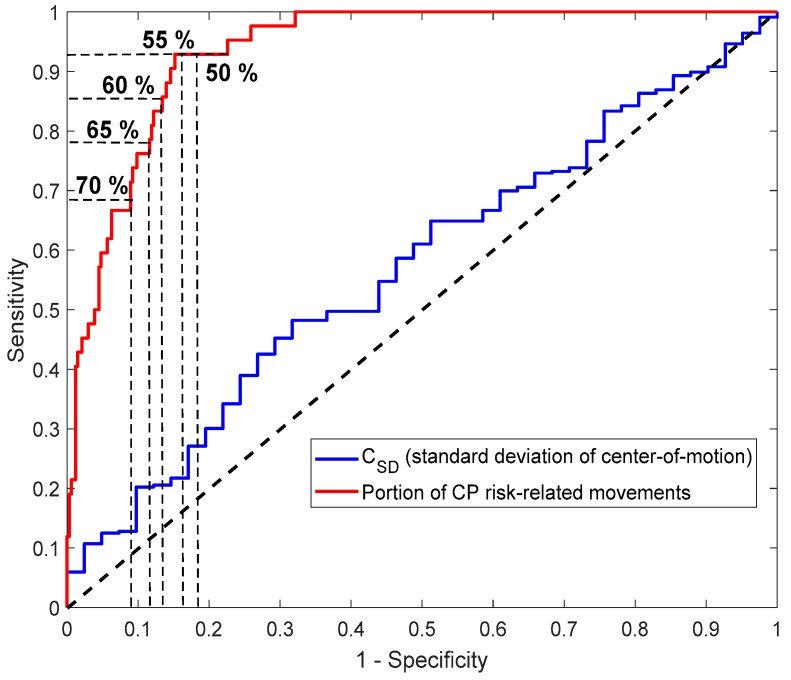
Receiver operating characteristic (ROC) curve of the proportion of periods with CP risk-related movements (red graph) compared to the ROC curve of the standard deviation of the center of motion (C_SD_) (blue graph). The horizontal and vertical dashed lines indicate points on the red ROC curve for the different decision thresholds represented in Table A2.

## Appendix A. Detailed Inclusion Criteria and Characteristics of the Multi-Site Cohort

The participants had previously participated in another study evaluating the assessment of general movements for the prediction of CP [19]. High-risk infants referred to follow-up at discharge from the NICU in three Norwegian and two US university hospitals were included. The inclusion criteria was at least one of the following for Norwegian sites 1–3: Infants referred to high-risk follow-up at discharge from the NICU based on at least one of the following: (a) GA < 28 and/or BW ≤ 1000 g (extremely low birth weight/extremely low GA; ELBW/ELGAN); (b) neonatal arterial ischemic stroke (NAIS); (c) neonatal encephalopathy (NE); (d) other significant risk factors for perinatal brain injury; site 4: Infants referred to high-risk follow-up at discharge from a quaternary NICU in the US with at least one of the following: (a) GA < 29 weeks; (b) congenital heart disease (CHD) in need of neonatal cardiac surgery; (c) medically complex infants including congenital anomalies and/or infants with syndromes/chromosomal abnormalities with an extended NICU stay beyond 10 weeks CA; (d) infants admitted to the NICU due to neurological symptoms and abnormal neonatal brain imaging; site 5: Infants born before 31 weeks GA with a birth weight below 1500 g who required oxygen at birth and were enrolled in a randomized controlled trial of two different doses of inhaled nitric oxide for neuroprotection (NOVA2 trial; https://clinicaltrials.gov/ct2/show/NCT00515281). Although one infant could have several risk factors for adverse neurodevelopment, all infants were classified into one risk group according to their main reason for referral. Extremely preterm infants (GA < 28 weeks and/or BW ≤ 1000 g) were classified as such irrespective of other risk factors, whereas preterm infants with GA 280–306 weeks and BW > 1000 g were classified as such only if they had no other risks of perinatal brain injury including moderate to severe imaging abnormalities. Demographic characteristics and primary reason for referral to follow-up risk assessment for the 377 included high-risk infants are shown in Table A1.

**Table A1:** Demographic variables and primary reason for referral to follow-up.

Risk Group	*N* (%)
GA < 28 weeks and/or BW ≤ 1000 g	167 (44.3)
- Boys	90 (53.9)
- GA (weeks), mean (SD)	26.3 (1.7)
- BW (g), mean (SD)	833 (178)
GA 28–36 weeks and BW > 1001 g	59 (15.6)
Neonatal arterial ischemic stroke	15 (4.0)
Neonatal encephalopathy	50 (13.3)
CHD w/surgery before 4 weeks	39 (10.3)
Other ^a^	47 (12.5)

BW = birth weight; GA = gestational age; CHD = Cardiac heart disease. Other ^a^: Infants who were referred to neurodevelopmental follow-up at discharge from the neonatal intensive care unit due to significant abnormalities on cerebral imaging (intraventricular hemorrhages III–IV, other intracranial hemorrhages with or without seizures, cystic periventricular leukomalacia, ventriculomegaly, venous sinus thrombosis), central nervous system infection, medically complex infants (syndromes/chromosomal abnormalities, multiple congenital anomalies, hydrops fetalis, severe lung hypoplasia, protracted hypoglycemia, seizures with unknown etiology) and severe intrauterine growth restriction. One second twin came to follow-up due to referral of the first twin.

## Appendix B. Feature Extraction of Complex and Variable Movements

The following multivariate empirical mode decomposition (MEMD) algorithm introduced by Rehman and Mandic [21] was used in the present study:

Step 1: Generate a Hammersley sequence-based point set on a *m*-1 dimensional sphere where *m* is the number of infant movement coordinates (*m* = 6 segments x 2 directions (x and y) = 12 in the present study).

Step 2: Compute the projection $p_{\theta_{k}}\left(t\right)$ of the infant movement dynamics x(t) (or residual **r**(*t*) or **d**(*t*) for iterative steps) along the unit direction vectors *θ_k_* of the *m*-dimensional sphere.

Step 3: Find the time instant $t_{\theta_{k}}$ corresponding to the maxima $p_{\theta_{k}}^{max}\left(t\right)$ of $p_{\theta_{k}}\left(t\right)$ along all *k* = 1, 2,…,*m*-1 dimensions.

Step 4: Obtain the envelope curves, $e_{\theta_{k}}\left(t\right)$, by component-wise spline interpolations between all time instants $t_{\theta_{k}}$ of $p_{\theta_{k}}^{max}\left(t\right)$.

Step 5: Compute the mean **m**(*t*) of all envelope curves, $e_{\theta_{k}}\left(t\right)$, across all *m* directions of the sphere by the following equation:

(A1) $$m\;\left(t\right)=\;\frac{1}{m-1}\overset{m-1}{\underset{k=1}{\sum }}e_{\theta_{k}}\left(t\right)$$

Step 6: The first series of details d_1_(*t*) around the mean m_1_(*t*) is defined as d_1_(*t*) = x(*t*) − m_1_(*t*). If d_1_(*t*) satisfies the selected stopping criteria, then d_1_(*t*) is defined as an intrinsic movement modality of the infant (i.e., intrinsic mode function; IMF) and Steps 2 to 5 is performed on first residual, r_1_(*t*) = x(*t*) − d_1_(*t*). The second IMF is defined as d_2_(*t*) = r_1_(*t*) − m_2_(*t*) with residual r_2_(*t*) = r_1_(*t*) − d_2_(*t*). Consequently, the *n*th IMF is defined as d*_n_*(*t*) = r*_n_*_−1_(*t*) − m_n_(*t*) with residual r*_n_*(*t*) = r*_n_*_−1_(*t*) − d*_n_*(*t*). This iterative shifting procedure (i.e., Steps 2 to 5) is continued until two maxima $p_{\theta_{k}}^{max}\left(t\right)$ of the projection $p_{\theta_{k}}\left(t\right)$ in Step 3 can no longer be found. If d*_n_*(*t*) does not satisfy the stopping criteria, Steps 2 to 5 are performed as an iterative procedure on d*_n_*(*t*) until the stopping criteria is met and an IMF is defined. Subsequently, Steps 2 to 5 are repeated on the residual, r*_n_*(*t*) = r*_n_*_−1_(*t*) − d*_n_*(*t*). The stopping criteria used in the present study is similar to the stopping criteria proposed by Rilling et al. [34], except that we excluded the criteria of equality between the number of zero crossings and number of maxima. The sum of all IMFs and the final residual, $x\left(t\right)=\overset{N}{\underset{n=1}{\sum }}d_{n}\left(t\right)+r_{N}\left(t\right)$ correspond to the infant segment movements x(*t*), where *N* is the number of IMFs. In the present study, *N* = 11 for all video recordings.

Step 7: A Hilbert–Huang transformation represents the infant segment movements as the real part of the following function:

(A2) $$x\left(t\right)=\overset{N}{\underset{n=1}{\sum }}a_{n}\left(t\right)e^{i\int^{\;}f_{n}\left(t\right)dt}$$

where the IMF of scale *n* is given by:

(A3) $$d_{n}\left(t\right)=a_{n}\left(t\right)e^{i\int^{\;}f_{n}\left(t\right)dt}$$

where a_n_(t) = [*a*_n,1_(*t*), *a_n,2_*(*t*),…,*a*_n,12_(*t*)] and f_n_(t) = [*f*_n,1_(*t*), *f_n,2_*(*t*),…,*f*_n,12_(*t*)] are the vector of instantaneous amplitude and frequency of d_n_(t) = [*d*_n,1_(*t*), *d_n,2_*(*t*),…,*d*_n,12_(*t*)] where each element is the instantaneous amplitude *a*_i_(*t*) and frequency *f*_i_(*t*) of *d*_n,i_(*t*) of a single segment in horizontal or vertical direction. The spectral density *S*_n,ii_ of *a*_n,i_(t) was estimated as:

(A4) $$S_{n,ii}\left(t\right)={\left|a_{n,i}\left(t\right)\right|}^{2}={\left|d_{n,i}\left(t\right)+iHd_{n,i}\left(t\right)\right|}^{2}$$

where H is the Hilbert matrix implemented according to the hilbert.m function in Matlab [35].

The instantaneous frequency *f*_n,i_(t) was estimated as:

(A5) $$f_{n,i}\left(t\right)=\frac{d\tan^{-1}\left(Hd_{n,i}\left(t\right)/d_{n,i}\left(t\right)\right)}{dt}$$

The time series *S*_n,ii_(*t*) and *f*_n,i_(*t*) were divided into 5 second non-overlapping time windows and the sum of *S*_n,i_(*t*) (i.e., total spectral energy) and mean f_n_(*t*) was computed for each window. This provided 264 features (i.e., number of IMFs x number of movement coordinates) for each time window. In addition, the instantaneous covariance *S*_n,ij_(*t*) for movement coordinates for body segment/direction *i* and *j* of each of the *N* scales was estimated as:

(A6) $$S_{n,ij}\left(t\right)=\left|a_{n,i}^{*}\left(t\right)\right||a_{n,i}\left(t\right)|=\left|d_{n,j}\left(t\right)-iHd_{n,j}\left(t\right)\right|\left|d_{n,i}\left(t\right)+iHd_{n,i}\left(t\right)\right|$$

*S*_n,ij_(*t*) was divided into 5 second non-overlapping time windows. The sum of *S*_n,ij_(*t*) (i.e., total spectral covariance) was assessed for each time window, resulting in 736 covariance combinations per time window. In total, 990 features were defined for each time window.

## Appendix C. CP Prediction Model

Let **X** be the scaled and centered feature matrix with 990 columns obtained by the procedure of Appendix B. Let **Y** be a column vector with −1/1 elements according to CP outcome (i.e., CP = 1 and non-CP = −1) for each 5 second time window where the length of **Y** is equal to the number of rows in X. The partial least square regression of **X** and **Y** was computed by the following nonlinear iterative partial least squares (NIPALS) algorithm [22]:

Step 1: X-weights **w** are defined by:

(A7) $$w=X^{T}u/u^{T}u$$

where Y-scores, **u** = **Y**, for the first iteration of Step 1 to 5.

Step 2: w is normalized according to its norm w=w/‖w‖

Step 3: X-scores t are defined by:

(A8) t=Xw

Step 4: Y-weights **c** are defined by

(A9) $$c=Y^{T}t/t^{T}t$$

Step 5: An updated set of Y-scores are defined by:

(A10) $$u=Yc/c^{T}c$$

Step 6: Step 1 to 5 is repeated until convergence for change in t is reached. The convergence criterion was set to $\frac{\left\|t_{old}-t_{new}\right\|}{\left\|t_{new}\right\|}<\varepsilon$, where $\varepsilon =10^{-8}$. If Step 1 to 5 was iterated 1000 times, the algorithm proceeds to Step 7 below.

Step 7: The first component (i.e., X-scores) of the PLS regression is defined as **t** of Equation A8. The first component is removed from **X** and **Y** by the following two equations:

(A11) $$Y=Y-tc^{T}$$

(A12) $$X=X-tp^{T}$$

where $p=X^{T}t/(t^{T}t)$

Step 8: Repeat Steps 1 to 7 until no more information is available in feature matrix **X** according to an inner 5-fold cross-validation procedure (see Section 2.2.3 in the main text). The obtained matrix **T** contains *N* columns for containing X-scores **t** in Equation A8 for each of the *N* repetitions of Steps 1 to 7.

The obtained matrix **T** defines the CP risk-related components of the original feature matrix **X** relevant for predicting CP outcome. Next, matrix **T** is an input in a linear discriminative analysis (LDA) to obtain a single score $Y_{est}$ in the range [−1, 1] for each 5 second window where $Y_{est}>0$ indicated a CP risk-related movement in the 5 second window. The following Bayesian approximation of LDA was used to define $Y_{est}$:

(A13) $$Y_{est}=TA\prime +a$$

where **A’** is all elements except the last element *a* in $A={\left(Z^{T}Z\right)}^{-1}Z^{T}B$, where B=1. Matrix **Z** is equal to [T,Y] for elements where **Y** > 0 and [−T,Y] for elements where Y≤0 (i.e., last column of **Z** equal to **Y**).

## Appendix D. Cross-Validation Procedure

The PLS-LDA (i.e., the CP prediction model) in Appendix C was optimized and validated by a double layer cross-validation procedure illustrated in Figure A1 below.

Step 1: The scaled and centered feature matrix X was divided into six folds where each, where the folds contained the movement features assessed in Appendix B for all 5 second epochs of seven infants with positive CP diagnosis and 56 infants with negative CP diagnosis. All 5 second epochs from one video/infant were only present in one of the folds.

Step 2: Five of the six folds were then used in an inner 5-fold cross-validation procedure to optimize the CP prediction model. In the inner cross-validation procedure, there were four folds for training and one fold for validation. The inner cross-validation loop was performed for each iteration of a backward feature selection procedure for all N number of components in the PLS regression. For each iteration of the backward feature selection procedure, the five folds of the inner cross-validation loop were repeated by selecting a new combination of infants/videos across the five folds of the inner cross-validation loop. The resulting optimal feature subset and selected number of components was the set producing the minimum mean square error (MSE) for the validation folds within the inner cross-validation loop. The difference between the MSE of the training and the validation set in the inner 5-fold cross-validation procedure was evaluated to ensure that overfitting did not occur.

Step 3: Second, the optimized CP prediction model was then tested on a sixth test fold in the outer 6-fold cross-validation procedure. The optimization procedure in Step 2 above was repeated for all folds in an outer cross-validation procedure. The test results in the present study for the CIMA model are represented for the outer 6-fold cross-validation procedure. This double cross-validation procedure prevents selection bias of movement features and the number of components of the PLS regression and increases the possibility of a final valid prediction result.

## Appendix E. ROC Curve and Decision Thresholds

The ROC curve in Figure A2 indicates that the performance of the CP prediction is dependent on the decision threshold (i.e., the percentage (%) of time periods with CP risk-related movements within the video recording). Table A2 shows that the specificity and, consequently, the PPV increases with a increasing decision threshold, but at the expense of a decrease in sensitivity and NPV (i.e., increase in the number of false negative decisions).

**Table A2:** Performance of CP prediction with different decision thresholds (%) for the proportion of periods with CP risk-related movements. The sensitivity, specificity, positive and negative predictive values and area under the curve (AUC) with 95% confidence intervals in brackets for the prediction of CP.

Threshold (%)	Sens. (%)	Spec. (%)	PPV (%)	NPV (%)
50	92.7 [80.1, 98.5]	81.6 [77.0, 85.5]	38.0 [32.5, 43.8]	98.9 [96.8, 99.6]
55	92.7 [80.1, 98.5]	83.9 [79.6, 87.7]	41.3 [35.2, 47.7]	99.0 [96.9, 99.6]
60	85.4 [70.8, 94.4]	86.6 [82.5, 90.1]	43.6 [36.6, 51.2]	98.0 [95.9, 99.0]
65	78.1 [62.4, 89.4]	89.3 [85.5, 92.4]	47.0 [38.5, 55.8]	97.1 [94.9, 98.4]
70	68.3 [51.9, 81.9]	92.3 [88.9, 94.9]	51.9 [41.3, 62.2]	96.0 [93.8, 97.4]

## Appendix F. Correlation between Imaging, GMA, and CIMA

Table A3, Table A4 and Table A5 indicate a weak correlation between imaging and CIMA, GMA and CIMA, and imaging and GMA.

**Table A3:** A weak correlation between imaging and CIMA.

Imaging/CIMA	CP Risk-Related Move > 50%	CP Risk-Related Move < 50%
**Abnormal MR**	42	42
**Normal MR**	57	234

Mean square contingency coefficient: *r* = 0.29.

**Table A4:** A weak correlation between GMA and CIMA.

GMA/CIMA	CP Risk-Related Move > 50%	CP Risk-Related Move < 50%
**Abnormal FMs**	44	42
**Normal FMs**	56	235

Mean square contingency coefficient: *r* = 0.30.

**Table A5:** A weak correlation between imaging and CIMA.

Imaging/GMA	Abnormal FMs	Normal FMs
**Abnormal MR**	35	49
**Normal MR**	50	241

Mean square contingency coefficient: *r* = 0.24.

## References

[1] Rosenbaum P., Paneth N., Leviton A., Goldstein M., Bax M., Damiano D., Dan B., Jacobsson B. (2007). A report: The definition and classification of cerebral palsy. Dev. Med. Child Neurol. Suppl..

[2] Oskoui M., Coutinho F., Dykeman J., Jette N., Pringsheim T. (2013). An update on the prevalence of cerebral palsy: A systematic review and meta-analysis. Dev. Med. Child Neurol..

[3] Novak I., Morgan C., Adde L., Blackman J., Boyd R.N., Brunstrom-Hernandez J., Cioni G., Damiano D., Darrah J., Eliasson A.C. (2017). Early, accurate diagnosis and early intervention in cerebral palsy: Advances in Diagnosis and Treatment. JAMA Pediatr..

[4] Guttmann K., Flibotte J., DeMauro S.B. (2018). Parental Perspectives on Diagnosis and Prognosis of Neonatal Intensive Care Unit Graduates with Cerebral Palsy. J. Pediatr..

[5] Baird G., McConachie H., Scrutton D. (2000). Parents’ perceptions of disclosure of the diagnosis of cerebral palsy. Arch. Dis. Child.

[6] Herskind A., Greisen G., Nielsen J. (2014). Early identification and intervention in cerebral palsy. Dev. Med. Child Neurol..

[7] Hadders-Algra M., Gramsbergen A. (2007). Discussion on the clinical relevance of activity-dependent plasticity after an insult to the developing brain. Neurosci. Biobehav. Rev..

[8] Maitre N. (2018). Skeptism, cerebral palsy, and the general movement assessment. Dev. Med. Child Neurol..

[9] Marcroft C., Khan A., Embleton N.D., Trenell M., Plotz T. (2014). Movement recognition technology as a method of assessing spontaneous general movements in high risk infants. Front. Neurol..

[10] Cabon S., Poree F., Simon A., Rosec O., Pladys P., Carrault G. (2019). Video and audio processing in paediatrics: A review. Physiol. Meas..

[11] Marchi V., Hakala A., Knight A., D’Acunto F., Scattoni M.L., Guzzetta A., Vanhatalo S. (2019). Automated pose estimation captures key aspects of General Movements at eight to 17 weeks from conventional videos. Acta Paediatr..

[12] Adde L., Helbostad J.L., Jensenius A.R., Taraldsen G., Grunewaldt K.H., Støen R. (2010). Early prediction of cerebral palsy by computer-based video analysis of general movements: A feasibility study. Dev. Med. Child Neurol..

[13] Rahmati H., Aamo O.M., Stavdahl Ø., Dragon R., Adde L. Video-based early cerebral palsy prediction using motion segmentation. Proceedings of the 2014 36th Annual International Conference of the IEEE Engineering in Medicine and Biology Society.

[14] Rahmati H., Martens H., Aamo O.M., Stavdahl Ø., Støen R., Adde L. (2016). Frequency Analysis and Feature Reduction Method for Prediction of Cerebral Palsy in Young Infants. IEEE Trans. Neural. Syst. Rehabil. Eng..

[15] Stahl A., Schellewald C., Stavdahl Ø., Aamo O.M., Adde L., Kirkerod H. (2012). An optical flow-based method to predict infantile cerebral palsy. IEEE Trans. Neural. Syst. Rehabil. Eng..

[16] Orlandi S., Raghuram K., Smith C.R., Mansueto D., Church P., Shah V., Luther M., Chau T. Detection of Atypical and Typical Infant Movement using Computer-based Video Analysis. Proceedings of the 2018 40th Annual International Conference of the IEEE Engineering in Medicine and Biology Society (EMBC).

[17] Kanemaru N., Watanabe H., Kihara H., Nakano H., Nakamura T., Nakano J., Taga G., Konishi Y. (2014). Jerky spontaneous movements at term age in preterm infants who later developed cerebral palsy. Early Hum. Dev..

[18] Hadders-Algra M. (2018). Neural substrate and clinical significance of general movements: An update. Dev. Med. Child Neurol..

[19] Støen R., Boswell L., de Regnier R.-A., Fjørtoft T., Gaebler-Spira D., Ihlen E., Labori C., Loennecken M., Msall M., Möinichen U.I. (2019). The predictive accuracy of the General Movement Assessment for cerebral palsy: A prospective, observational study of high-risk infants in a clinical follow-up setting. J. Clin. Med..

[20] Rahmati H., Dragon R., Aamo O.M., Adde L., Stavdahl Ø. (2015). Weakly supervised motion segmentation with particle matching. Comput. Vis. Image Underst..

[21] Rehman N., Mandic D.P. (2010). Multivariate empirical mode decomposition. Proc. R. Soc. A.

[22] Huang N.E., Shen Z., Long S.R., Wu M.C., Shih H.H., Zheng Q., Yen N.C., Tung C.C., Liu H.H. (1998). The empirical mode decomposition and the Hilbert spectrum for nonlinear and non-stationary time series analysis. Proc. R. Soc. Lond. Ser. A Math. Phys. Eng. Sci..

[23] Cans C. (2000). Surveillance of cerebral palsy in Europe: A collaboration of cerebral palsy surveys and registers. Surveillance of Cerebral Palsy in Europe (SCPE). Dev. Med. Child Neurol..

[24] Wold S., Sjöström L., Erikson L. (2001). PLS regression: A basic tool of chemometrics. Chemom. Intell. Lab. Syst..

[25] Tang L., Peng S., Bi Y., Shan P., Hu X. (2014). A New Method Combining LDA and PLS for Dimension Reduction. PLoS ONE.

[26] Einspieler C., Prechtl H.F., Bos A., Ferrari F., Cioni G. (2004). Prechtl’s Method on the Qualitative Assessment of General Movements in Preterm, Term and Young Infants.

[27] Einspieler C., Peharz R., Marschik P.B. (2016). Fidgety movements—Tiny in appearance, but huge in impact. J. Pediatr. (Rio J).

[28] Palisano R.J., Rosenbaum P., Walter S., Russell D., Wood E., Galuppi B. (1997). Development and reliability of a system to classify gross motor function in children with cerebral palsy. Dev. Med. Child Neurol..

[29] Palisano R.J., Hanna S.E., Rosenbaum P.L., Russell D.J., Walter S.D., Wood E.P., Raina P.S., Galuppi B.E. (2000). Validation of a model of gross motor function for children with cerebral palsy. Phys. Ther..

[30] Støen R., Songstad N.T., Silberg I.E., Fjørtoft T., Jensenius A.R., Adde L. (2017). Computer-based video analysis identifies infants with absence of fidgety movements. Pediatr. Res..

[31] Einspieler C., Bos A.F., Krieber-Tomantschger M., Alvarado E., Barbosa V.M., Bertoncelli N., Burger M., Chorna O., Del Secco S., DeRegnier R.A. (2019). Cerebral Palsy: Early markers of clinical phenotypes and functional outcome. J. Clin. Med..

[32] Cao Z., Hidalgo G., Simon T., Wei S.-E., Sheikh Y. (2018). OpenPose: Realtime multi-person 2D pose estimation using part affinity fields. arXiv.

[33] Fjørtoft T., Einspieler C., Adde L., Strand L.I. (2009). Inter-observer reliability of the Assessment of Motor Repertoire 3 to 5 Months based on video recordings of infants. Early Hum. Dev..

[34] Rilling G., Flandrin P., Gonçalves P. On empirical mode decomposition and its algorithms. Proceedings of the IEEE-EURASIP, Workshop on Nonlinear Signal and Image Processing NSIP-03.

[35] Marple S.L. (1999). Computing the Discrete-Time Analytic Signal via FFT. IEEE Trans. Signal Process..

